# Bridging vision and touch: advancing robotic interaction prediction with self-supervised multimodal learning

**DOI:** 10.3389/frobt.2024.1407519

**Published:** 2024-09-30

**Authors:** Luchen Li, Thomas George Thuruthel

**Affiliations:** Department of Computer Science, University College London, London, United Kingdom

**Keywords:** predictive learning, self-supervised learning, physical robotic interaction, information fusion and compression, multi-modal sensing

## Abstract

Predicting the consequences of the agent’s actions on its environment is a pivotal challenge in robotic learning, which plays a key role in developing higher cognitive skills for intelligent robots. While current methods have predominantly relied on vision and motion data to generate the predicted videos, more comprehensive sensory perception is required for complex physical interactions such as contact-rich manipulation or highly dynamic tasks. In this work, we investigate the interdependence between vision and tactile sensation in the scenario of dynamic robotic interaction. A multi-modal fusion mechanism is introduced to the action-conditioned video prediction model to forecast future scenes, which enriches the single-modality prototype with a compressed latent representation of multiple sensory inputs. Additionally, to accomplish the interactive setting, we built a robotic interaction system that is equipped with both web cameras and vision-based tactile sensors to collect the dataset of vision-tactile sequences and the corresponding robot action data. Finally, through a series of qualitative and quantitative comparative study of different prediction architecture and tasks, we present insightful analysis of the cross-modality influence between vision, tactile and action, revealing the asymmetrical impact that exists between the sensations when contributing to interpreting the environment information. This opens possibilities for more adaptive and efficient robotic control in complex environments, with implications for dexterous manipulation and human-robot interaction.

## 1 Introduction

In contemporary neuroscience, it has been widely acknowledged that humans rely on the predictive processing (PP) mechanism to perform complex interaction tasks (Friston, 2005). When perceiving and interacting with the dynamic physical and social surroundings, the brain builds and maintains internal predictive models based on sensorimotor signals, from basic predictions of the agent’s own actions (i.e., active inferences) to high level perceptual inference that predicts the outcomes of the external world (Köster et al., 2020). In order to generate adaptive and appropriate behaviors in the highly variable and uncertain real world, the internal models are continuously updated by comparing the incoming sensory signals with the predictions to minimize the error (Nagai, 2019).

Therefore, the multiple sensory signals play a key role in building the agent’s interaction perception. While vision is considered in public thinking to dominate perceptual experience, other sensation can also be pivotal in certain situations. Neurophysiology studies have proven that instead of functioning in isolation, different human sensory modalities are interconnected (Stein and Meredith, 1993; Shams and Kim, 2010). For example, visually impaired individuals may recognize shapes through auditory sensory substitution (Amedi et al., 2007), and touch sensing can complement vision in object processing tasks with its shared object representation (James et al., 2007). This integration and cross-modality influence of different sensing modalities are also essential for multi-sensory predictive learning, which can be observed in early cognitive development of humans (Nagai, 2019).

In robotics, it is also a fundamental challenge to accurately predict the consequences of the agent’s actions on its environment, especially in real-world physical interactions characterized with increasing complexity and stochasticity. In light of the advancements in deep learning and computer vision, the robotic interaction tasks are commonly performed with video prediction models based on deep neural networks (Oprea et al., 2020), which has been widely applied in autonomous driving, visual servoing manipulation and grasping, etc. Many works leverage Long Short-Term Memory (LSTM) networks to effectively extract temporal dependencies in the observed video sequences for prediction of future frames (Srivastava et al., 2015; Lotter et al., 2016). In addition, video predictive models conditioned on action received broader applications, such as reinforcement learning (Escontrela et al., 2024; Lenz et al., 2015), motion planning (Sarkar et al., 2019; Zang et al., 2022), etc.

Driven by the development of sensor technologies, an increasing academic interest shifts to tactile sensing for its crucial role in more adaptive, robust and dexterous robotic manipulation. Compared to vision that contributes to broad scene understanding and initial localization, tactile feedback excels in providing contact details during interaction such as textures, contact forces, mass, stiffness, and other invisible physical properties (Yousef et al., 2011). Therefore, a number of studies work on learning tactile-based predictor for various uses, including slip control (Dong et al., 2019; Donlon et al., 2018; Nazari et al., 2023; Zhang et al., 2023), pose estimation (Bauza et al., 2019; 2023; Kelestemur et al., 2022), in-hand manipulation (Yang et al., 2023; Lepert et al., 2023; van Hoof et al., 2015), etc.

However, the aforementioned approaches based on single sensory modality may lead to restricted perception capabilities and increasing prediction error. Therefore, inspired by the multi-modal cognition system for human, some researchers investigate the combination of vision and touch through cross-modality translation. Lee et al. (2019) proposed a conditional generative adversarial networks to generate realistic translation between material texture image and vision-based surface tactile information. Li et al. (2019) achieved cross-modality prediction between vision and touch through a ResNet based adversarial learning framework, which aims to synthesize plausible sensory signals from another sensation. Other attempts have been made to utilize the integrated visuo-tactile representation for object recognition and classification (Falco et al., 2017; Sun et al., 2016; Castellote López, 2023). Nevertheless, the exploration of vision-tactile integration in robotic interaction is still in its infancy. While the aforementioned studies are mainly conducted in static scenarios, predictive models that are able to capture the connections between vision and touch in the dynamic settings of physical interactions have not been sufficiently investigated.

This work aims to explore the interconnection and cross-modality influence between vision and tactile sensation in the scenario of dynamic robotic interactions. While visual feedback contains global perception about the semantic and geometric properties of the scene, it also comes with the inherent limitations with occlusions and lighting variations. Likewise, tactile sensation provides insights into texture, pressure, deformation, and other contact-related local information, but it may be inferior in general object localization. These two sensing modalities may complement each other towards more comprehensive sensory perception required for complex physical interactions. Therefore, we introduce a multi-modal fusion mechanism into the well-known video prediction architecture Convolutional Dynamic Neural Advection (CDNA) model (Finn et al., 2016), which is specifically designed for predicting the visual outcomes of robotic manipulations through pixel-level motion modelling. By revising the model to accommodate both vision and tactile sensation, we investigate the cross-modal connection between them through a series of prediction tasks within the context of robot-object physical interaction, where the predictive model is supposed to capture the dynamic transformation between the frames over timesteps, given the known robot action and vision-tactile information. We believe the insightful results reveal potential in advancing robotic perception in interactive tasks such as dexterous manipulation, deformable object manipulation and human-robot interaction, etc.

The contributions of this work are as follows. In this article, we present a novel advancement in the predictive learning of robotic interaction by enhancing the Convolutional Dynamic Neural Advection (CDNA) framework into a multi-sensory action-conditioned prediction model. The primary sensory input is combined with the auxiliary sensory input in the latent space before fed into the prediction modules, which complements the single-modality prototype with more comprehensive perception information. A series of multi-modal fusion architectures have been tested to optimize the model’s capability to assimilate information across vision and tactile sensation to assist the investigation into the cross-modal connection. In addition, the compressed multi-modal representation in the latent space is valuable for applications beyond prediction tasks, which could be leveraged in control systems to develop more efficient and adaptive control strategies. Compared to the static analysis between vision and touch in Lee et al. (2019); Li et al. (2019), our work focuses on connecting the multiple sensory inputs in a more dynamic setting. We build a robotic system to collect the vision-tactile pairs along with the action data during robot-object interaction tasks, specifically involving a combination of sliding and rolling motions. This process generates a multi-modal robotic interaction dataset that includes sequences of both vision and tactile sensory information, along with the corresponding robot position configurations. As there lacks sufficient open dataset for robot interaction that contains sensing modality beyond vision (Dasari et al., 2019), the proposed dataset could be beneficial to future study towards more enriched robotic perception with complex and dynamic environments. We present a series of comparative studies to explore the interconnection between vision and touch during robotic interaction predictions, performing experiments under various sensory combinations (single modality vs. vision-tactile integration) and different environmental conditions (limited vision vs. full vision) to investigate how these factors influence prediction accuracy and the robustness of the model. Both qualitative and quantitative analysis are presented to evaluate the performance. Through predictions of one sensory input conditioned on another sensation, we reveal the asymmetrical influence that vision and touch exert on each other. These findings highlight the distinct roles these sensory modalities play during interactions, as well as the synergistic effect that emerges when they are combined. In addition, as another significant factor in interaction, the impact of robot action data is also evaluated, which reveals the mutual relationship between the sensory observations and the environment’s dynamics.

## 2 Materials and methods

### 2.1 Problem formulation

The objective of our work is to investigate the connection between the multi-modal sensory inputs in the predictive learning of robot interaction dynamics. Our prediction model is based on the Convolutional Dynamic Neural Advection (CDNA) framework proposed by Finn et al. (2016), which is originally designed for action-conditioned video prediction. By introducing a multi-modal fusion mechanism, we expand the vision-to-vision prediction into more comprehensive prediction tasks that aim to predict future frames (either vision or tactile) of a robot’s interaction with its environment utilizing a combination of vision (V), tactile (T) and action (A) data. Here we present the formulation of tactile-conditioned vision prediction, the same thing also applies to tactile prediction conditioning on vision.

Given (i) a set of past vision frames $V_{t-m+1:t}=\left\{V_{t-m+1},\ldots ,V_{t}\right\}$ , where t is the current timestep and m is the number of context frames considered during prediction and (ii) a set of conditioning frames of tactile $T_{t-m+1:t+n}=\left\{T_{\text{t}-m+1},\ldots ,T_{t+n}\right\}$ and action $A_{t-m+1:t+n}=\left\{A_{\text{t}-m+1},\ldots ,A_{t+n}\right\}$ , where n is the number of future frames to be predicted, the prediction model F can be defined in Equation 1:

$$F\left(V_{t-m+1:t},T_{t-m+1:t+n},A_{t-m+1:t+n};\theta \right)={\hat{V}}_{t+1:t+n}$$ (1)

where $\hat{V}$ denotes the predicted vision sequences, and θ represents the parameter of the predictive model.

Therefore, the objective function L aims to minimize the pixel-wise difference between the predicted and actual future vision frames over the n time steps as detailed in Equation 2:

$$L=\min\overset{i=t+n}{\underset{i=t+1}{\sum }}D\left({\hat{V}}_{i},V_{i}\right)$$ (2)

where D is the loss function that measures the discrepancy between the prediction and ground truth in pixel space. In our work, we choose mean squared error (MSE) loss: $L={\left\|{\hat{V}}_{i}-V_{i}\right\|}^{2}$ .

### 2.2 Multi-modal sensor fusion for robotic interaction prediction

#### 2.2.1 Model overview

**FIGURE 1:**
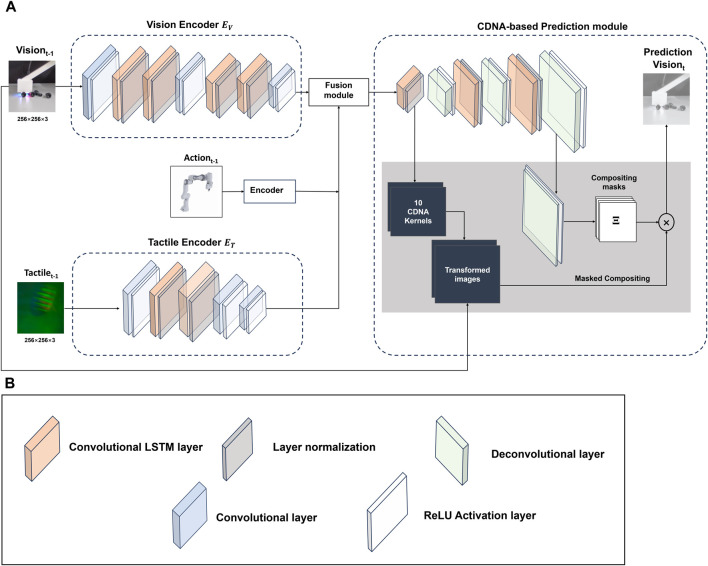
Architecture of the prediction model: vision and tactile data are encoded through a attached Conv-LSTM based network. The multi-modal fusion module combines the vision, tactile and reshaped action data in the latent space, which consists of a layer of Conv-LSTM and an attention mechanism. The prediction is based of the CDNA mechanism, which models pixel-wise transformation with a series of transformation kernels. **(A)** Model overview: here the architecture of the tactile-conditioned vision prediction model is presented, which is applicable to the tactile prediction as well. **(B)** Details of the building blocks: the Conv-LSTM layer is followed by layer normalization to stabilize the training. The outputs of both convolution and transposed convolution are activated by the Rectified Linear Unit (ReLU) functions.

The overview architecture of the predictive model in this work is illustrated in Figure 1. The Convolutional Dynamic Neural Advection (CDNA) framework is chosen as the baseline model from which our multi-modal prediction system is built from, the auto-encoder structure of which enable the model to work in a self-supervised paradigm, using unlabeled raw sensor data to learn the dynamics about robotic physical interaction. Here we present the architecture of tactile-conditioned vision prediction. For tactile prediction conditioned on vision data, we switch position of these two modalities while remaining the rest of the framework. The predictive model consist of three main modules: (i) individual modality modules that extract distinct features from the vision, tactile and action sensory input into the latent space. (ii) multi-modal sensory fusion module that learn a compressed shared representation based on the latent features in different modalities. (iii) CDNA-based prediction module that performs motion prediction by modelling pixel-wise transformation over time.

#### 2.2.2 Individual modality module

The individual modality modules aim to encode the raw sensory inputs into latent features through deep neural networks, during which the high-resolution spatial data is compressed into the more compact latent representations. This data compression technique effectively captures the underlying patterns of the input data, as well as reducing the computational costs during the model training (Mahendran and Vedaldi, 2015; Han et al., 2015). More importantly, the sensory data of different modalities are mapped into a shared latent space, which facilitates the subsequent multi-modal fusion.

Given the vision and tactile frame at the previous timestep $V_{t-1}\in R^{\mathrm{256\times 256\times 3}}$ and $T_{t-1}\in R^{\mathrm{256\times 256\times 3}}$ , latent features are extracted by the corresponding encoders for each modality, formulated as Equation 3:

$$\begin{array}{cc} & f_{T,t-1}=E_{T}\left(T_{t-1}\right)\\ & f_{V,t-1}=E_{V}\left(V_{t-1}\right) \end{array}$$ (3)

where $E_{T}$ , and $E_{V}$ denote the encoders with respect to vision and tactile.

As illustrated in Figure 1, both $E_{T}$ , and $E_{V}$ are constructed with stacked layers of CNNs and Convolutional Long Short-Term Memory (Conv-LSTMs). Followed by the ReLU activation layers, the CNNs mainly serve to compress the feature as well as extract the spatial information from the frames. Compared to the standard LSTM architecture, the Conv-LSTMs employed in the model replace the internal fully connected gate operations with convolution, which can capture both temporal dynamics and spatial dependencies within the sequential data (Shi et al., 2015). To reduce the computation burden of the model, $E_{T}$ employs a lighter architecture of Conv-LSTMs (two layers with 32 and 64 filters respectively) than $E_{V}$ (four layers with 32, 32, 64 and 64 filters respectively), which is sufficient as the tactile sensory fulfill its complementary role in the vision prediction tasks. After encoding, both vision and tactile features are compressed to the same spatial size of 64×32×32 .

#### 2.2.3 Multi-modal sensory fusion module

**FIGURE 2:**
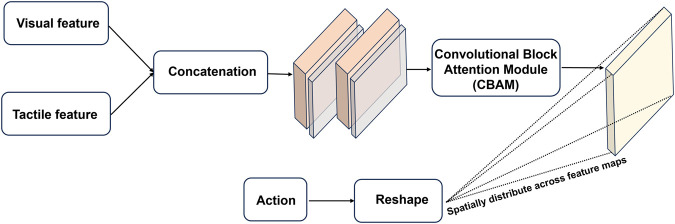
Architecture of the multi-modal sensory fusion module: the latent features of the vision and tactile sensation are concatenated along the channel dimension before fed into two layers of Conv-LSTM that extract the spatial and temporal dynamics from the combined features. An attention block is incorporated to reweigh the feature values, thereby enabling the model to prioritize the most relevant information for accurate prediction. To integrate robot action information, the raw action data is reshaped and broadcasted across the spatial extent of the vision-tactile feature map, ensuring an aligned and cohesive representation for further processing and combination.

While both vision and tactile data are represented as image sequences, there is still inherent modality difference between them. In latent space, the compressed feature for each modality contains the distinct characteristics of the original sensory source, and simple concatenation is insufficient to obtain complementary information capture the complementary information they provide. Therefore, it is crucial to use a proper fusion mechanism which is able to resolve the heterogeneity as well as efficiently extract the correlation between vision and touch. Figure 2 illustrated the internal framework of the multi-modal fusion module, which takes the latent values of vision and touch as input to learn a shared representation of two sensory modalities, as well as combining robot action data to form the integrated features for prediction. The vision and tactile data are concatenated in the channel dimension before fed into a layer of Conv-LSTM followed by a cross-modality attention block. Compared to simple concatenation, the use of Conv-LSTM reserves the spatial-temporal dynamics of the integrated data, which is crucial for handling the sequential data. In addition, we use the Convolutional Block Attention Module (CBAM) that generates attention maps along both the channel and spatial dimensions before multiplied with the input (Woo et al., 2018). The attention mechanism applied to the combined vision-tactile features enhances the model performance as it allows the model to selectively focus on the information that is more relevant to the formulated prediction problem, enabling more effective representations of the multi-modal features. Unlike vision and tactile data, the raw data of robot action is a 1-dimension vector that contains six position values related to the end-effector. Therefore, preprocessing is required to match the unaligned sensory data in different domains. The action data is first expanded to contain two extra dimensions as the vision-tactile data. Then the reshaped action data is broadcasted across the spatial extent of the vision-tactile feature map before concatenation in the channel dimension (which is achieved by replicating the action values along the newly introduced dimensions to ensure compatible size with the vision-tactile features). The combined feature is then fed in to a CNN layer to be reweighted with learnable coefficient.

#### 2.2.4 Convolutional dynamic neural advection (CDNA) based prediction module

Originally proposed by Finn et al. (2016), the CDNA-based prediction module explicitly models the pixel motions between frames through a set of transformation kernels. Compared to video prediction approaches that either reconstruct the entire future frames from scratch or focus on the implicit internal state (Srivastava et al., 2015; Mathieu et al., 2015; Oh et al., 2015), CDNA provides a more efficient and accurate motion prediction, especially in robotic manipulation tasks with deterministic motion patterns. As illustrated in Figure 1, the output of the multi-modal fusion module $f_{c,t-1}$ is fed into two network branches, one is for predicting a set of transformed vision frames based on the previous frame and the learnable transformation kernels, the other is for generating the corresponding masks that weight the impact of different transformation estimations on the final prediction. In order to generate the transformation kernels, the combined feature $f_{c,t-1}$ is fed to a fully connected layer denoted as FC before being reshaped into the desired dimensions, as detailed in Equations 4, 5:

$$K^{n}=\mathrm{reshape}\left(FC\left(f_{c,t-1}\right)\right)$$ (4)

$${K^{\prime }}^{n}=\frac{K^{n}}{\sum K^{n}}$$ (5)

where $K^{n}$ , n = 1,2, ⋅ ,N, denotes the transformation kernels, each kernel is of size m×m (in this work, N = 10 and m = 5), ${K^{\prime }}^{n}$ is the normalized kernels.

The normalized kernels are then applied to the previous vision frame $V_{t}-1$ to predict a set of transformation images for each pixel (x,y) , as shown in Equation 6:

$$J_{t}^{n}\left(x,y\right)=\underset{i\in \left(-m,m\right)}{\sum }\underset{j\in \left(-m,m\right)}{\sum }{K^{\prime }}^{n}\left(i,j\right)V_{t-1}\left(x-i,y-j\right)$$ (6)

where $J_{t}^{n}$ , n = 1,2, ⋅ ,N denotes the transformation images.

On the other hand, the masks that weight the transformation images $\Xi_{c}$ , c = 1,2, ⋅ ;,N+1, are obtained by applying a channel-wise softmax to the output of another branch of the network, which contains Conv-LSTMs interspersed with deconvolutional layers. The original input vision frame is appended into the transformation images to match the extra mask channel that corresponds to static background. Therefore, the final predicted vision frames ${\hat{V}}_{t}$ is obtained by compositing the masks and transformed images using Equation 7:

$${\hat{V}}_{t}=\underset{c}{\sum }{\hat{J}}_{t}^{c}⊙\Xi_{c}$$ (7)

where c denotes the channel and ⊙ refers to the element-wise multiplication.

### 2.3 Experiment setup

**FIGURE 3:**
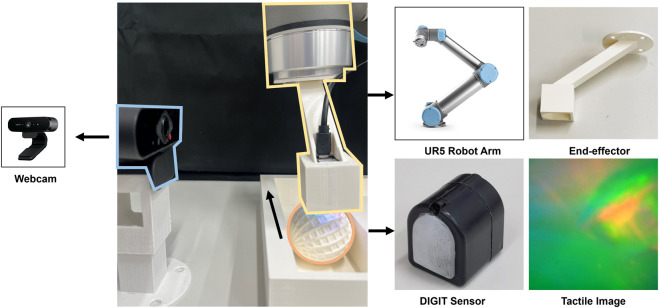
Experiment setup: the interaction system consists of a UR5 robot arm that is equipped with a DIGIT tactile sensor to collect the contact information. A webcam is set aside to capture the visual scene of the physical interactions. The graphs on the right show the details of the 3D-printed end-effector and the sample images of the vision-based tactile sensor.

In order to accomplish the predictive tasks of interaction, we build a robotic system with multi-modal perception, as shown in Figure 3. Here we describe the hardware setting of the system, the data collection procedure, and the vision-tactile dataset of robot-object interaction built for training and testing the model that will be elaborated in the next section.

#### 2.3.1 Hardware setting

As illustrated in Figure 3, a Universal Robots UR5 robot arm is equipped with a 3D-printed end-effector to automatically interact with the objects. A webcam is set up with support at the side of the arm to capture videos of interaction scenes. In addition, we mount a tactile sensor called DIGIT (Lambeta et al., 2020) to the holder on the end-effector for collecting the raw tactile sequences. DIGIT is an optical-based tactile sensor that captures fine details of the contact surface at high spatial resolution that surpasses other types of tactile sensors, such as resistive-based (Choi, 2010; Kim et al., 2009; Zhang and Miki, 2010), capacitive-based (Lee et al., 2008), piezoelectric-based sensors (Zhang et al., 2006; Noda et al., 2006; 2009), etc. The surface of DIGIT is a soft, transparent silicone gel that deforms upon contact, underneath which is a camera that captures the deformation patterns of the gel with an array of LEDs illuminating the sensing field. This specific design allows DIGIT to generate three-channel tactile images about the texture and geometry of the contact surface, which benefits the multi-modal fusion of vision and touch by avoiding the obstacle in developing network architectures caused by the modality difference. Additionally, compared to another type of vision-based tactile sensor Gelsight (Yuan et al., 2017), DIGIT provide a more compact and low-cost solution with a trade-off in resolution and sensitivity. However, with an image resolution of $640^{*}$ 480 sufficient for prediction tasks and greater ease of integration in the robotic system, DIGIT is the optimal choice for our work.

#### 2.3.2 Data collection

The UR5 robot arm is connected to a PC device and controlled through the URScript in Python. During the data collection, the robotic system is designed to perform a series of straight-line object interaction trials. To accomplish this, the initial position of the end-effector is adjusted to allow the contact surface of the tactile sensor to be close above the object, and then the robot arm is controlled to move horizontally along either X or *Y*-axis for a certain distance in each trial. In this way, there is continuous contact between the tactile sensor and the object, providing efficient physical interaction throughout the motion of the robot. We collected the synchronized data of vision, tactile and action at a sample rate of 20 frames per second, in which both vision and tactile data are stored as image sequences, and the action data used in this work is the real-time position of the robot arm’s end-effector at each sampled time step.

#### 2.3.3 Object interaction dataset

The dataset consists of 250 interaction trials of different objects, including screws of 100 trials, ball with distinct textures in three sizes (50 trials each size). In the tasks explored, the robot interacts with objects by applying forces that induce both sliding and rolling motions. The interactions are carefully controlled to ensure they are conducted in a contact-rich manner, maintaining consistent contact between the gel surface of the tactile sensor and the object. Due to the relative motion between the robot and the object, combined with the complex dynamics involved, these interactions provide an ideal setting for investigating multi-modal sensing. In such scenarios, relying on a single modality may not fully capture the critical aspects of the interaction, highlighting the importance of integrating multiple sensory inputs. Each trial contains 40 frames of synchronized vision, tactile and action data (position of the end-effector), in which the vision and tactile frames are preprocessed into RGB images with a size of 256 × 256 × 3. In order to augment the data with diversity, the position of the webcam has been moved in different trials to generate a diverse viewpoint. In addition, the motion parameters (velocity, acceleration, distance) that control the movement of the robot arm and the initial contact points are adjusted in different trials to create a more diverse set of interaction scenarios.

#### 2.3.4 Screw interaction subset

For experiments in this work, a subset of the above object interaction dataset has been used for computation efficiency, which contains 60 interaction trials of screws. The screw dataset has been split into a training dataset of 40 trials, a validation dataset of 15 trials and a test dataset of 5 trials. Each trial within the dataset has been trimmed into a series of sequences with 15 frames, generating sufficient samples for training the model.

## 3 Results

In this section, we aim to evaluate the impact of integrating multi-modal sensory input during physical robot interactions with objects, investigating the cross-modality connection and influence between vision and touch in the scenario of robotic predictive learning. Therefore, based on the experiment setup introduced in 2.2, we perform a series of prediction trials that compare the proposed multi-modal prediction system with baseline models that remove certain sensation. More specifically, we compare the tactile-conditioned vision prediction models with its tactile-excluded counterpart to investigate the significance of tactile sensation. Same experiments are conducted for tactile prediction model conditioning on visual sensing. In addition, the contribution of action data is also explored by comparative analysis between the aforementioned models and their non-action counterparts. Both qualitative and quantitative evaluation are conducted to provide more comprehensive insights.

### 3.1 Implementation details

We test the models on the aforementioned screw interaction dataset, which contains 60 interactive trials, each of which consists of 40 frames of synchronized vision, tactile and action data. Each interactive trials are divided in sequences of 15 frames, generating 1,500 interaction sequences in total. Before fed into the model, the vision and tactile images are resized to $R^{\mathrm{256\times 256\times 3}}$ . The Models are developed and trained in PyTorch on Nvidia RTX A1000 GPU’s with an initial learning rate of 0.001.

#### 3.1.1 Evaluation metrics

The quantitative evaluations are performed using two metrics. Mean Absolute Error (MAE) is used to measure the discrepancy between the predicted frames and the ground truth at the pixel level. The other metric is Structural Similarity (SSIM) that evaluates changes in structural information, luminance, and contrast between the predicted and actual images (Wang et al., 2004). The SSIM index given by Equation 8 can vary between −1 and 1, where a value of 1 indicates perfect similarity:

$$\mathrm{SSIM}\left(x,y\right)=\frac{\left(2\mu_{x}\mu_{y}+c_{1}\right)\left(2\sigma_{xy}+c_{2}\right)}{\left(\mu_{x}^{2}+\mu_{y}^{2}+c_{1}\right)\left(\sigma_{x}^{2}+\sigma_{y}^{2}+c_{2}\right)}$$ (8)

where x and y denote the windowed sections of the predicted and actual images, respectively; $\mu_{x}$ and $\mu_{y}$ are the average pixel values; $\sigma_{x}^{2}$ and $\sigma_{y}^{2}$ refer to the variances; $\sigma_{xy}$ denote the covariance; $c_{1}$ and $c_{2}$ are constants used to stabilize the division.

### 3.2 Architecture comparison

In order to optimize the multi-modal sensory fusion, three architectures of the fusion modules are evaluated: (a) simple concatenation between vision and tactile data; (b) apply a layer of CNN after concatenation; (c) the proposed architecture in 2.3.3 that employs Conv-LSTM as well as a multi-modal attention mechanism. The architectures are tested on a subset of the screw interaction dataset, which contains 30 interaction trials and are divided into a training, validation and test set with 18, 8 and 4 trials respectively. Table 1 shows the quantitative results of different architecture in the tactile-conditioned vision prediction task, including the average MAE and SSIM over the prediction horizon of 14 time steps.

**TABLE 1:** Average tactile-conditioned vision prediction performance of different fusion architecture: baseline 1 refers to fusion through simple concatenation, baseline 2 refers to fusion with CNN. The proposed fusion mechanism outperforms the baseline models in terms of both MAE and SSIM scores.

Model	Baseline1	Baseline2	Proposed model
MAE ↓	0.0143	0.0141	**0.0123**
SSIM ↑	0.9677	0.9663	**0.9687**

Bold values indicate the best performance for each metric across the models compared.

Table 1 shows that while the use of CNN improves the model performance, the proposed model equipped with Conv-LSTM and attention mechanism performs best over the whole prediction time range. Therefore, the following evaluations are implemented with the proposed fusion architecture.

### 3.3 Hyper-parameter selection

As stated in 2.1, the prediction of future frames is based on the current frame and a set of context frames. In implementations, the model performs sequential prediction by using the frame from the previous time step as its input, and then iteratively generating predictions for multiple future frames. The context frames are introduced during the training process, but not for validation and testing. Given a specific number of context frames N, the prediction of the first N frames is based on the ground truth data, while the prediction afterwards will take the predicted results as the input. Therefore, the number of context frames is a hyper-parameter that might affect the performance of the model. Table shows the performance of models trained with various selection of context numbers in tactile-conditioned vision prediction task, the screw subset used in 3.2 serves as the training dataset in this case as well.

It can be observed in Table 2 that the prediction performance peaks with 4 context frames. While the employment of historic frames assist the model in capturing the temporal dynamics, they might also cause overfitting beyond certain point. Therefore, we have selected the contextual frames of 4 in the training stage, which not only ensures the production of reasonable results but also grants the model sufficient autonomy for independent inference, achieving a balance between providing adequate historical context and maintaining the model’s predictive capabilities.

**TABLE 2:** Average tactile-conditioned vision prediction performance of models with different number of context frames: the prediction performance over both MAE and SSIM metrics improves with the increasing context frames, and the context frame number of 4 is selected to be used in this paper considering the total sequence length.

Number of context frames	1	2	3	4	5
MAE ↓	0.0512	0.0265	0.0173	0.0123	0.0135
SSIM ↑	0.8691	0.9304	0.9571	0.9687	0.9623

### 3.4 Action-conditioned interaction prediction combining vision and tactile

#### 3.4.1 Tactile-conditioned vision prediction

**FIGURE 4:**
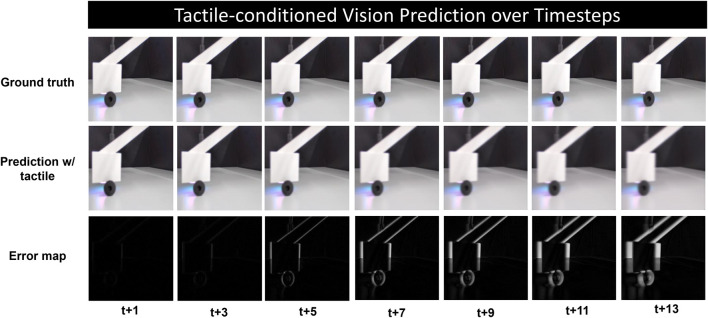
Qualitative results of tactile-conditioned vision prediction over long time horizon, the ground truth and predicted frames are shown at every three time steps from t+1 to t+13, the last row depicts the error map that show the prediction error more explicitly.

In this section, we explore the action-conditioned vision prediction of physical robot interaction with the inclusion of tactile sensation, which is supposed to complement the system’s perception with contact-related local information. Figure 4 shows the qualitative results over time steps, the error maps between ground truth and predicted frames are calculated to obtain a more explicit observation of the discrepancy. It can be seen that prediction quality of the visual scene degrades over time. While the model manages to predict the motion clearly in the first few frames, the results become blurry as the uncertainty increases over the time range.

From the error map, it can be observed that the prediction error is more salient at the edge of the moving robot and object, while the static background is more accurately constructed. In addition, the blur occurs across the location gap between the ground truth and prediction, which indicates the model’s effort in capturing the dynamics of the system. This blurring effect, especially noticeable in the direction of movement, underscores the model’s partial success in recognizing and following the correct motion trajectory. However, it also reflects the model’s limitations in precisely rendering the motion details over long time steps, resulting in a less clear depiction of dynamic interactions within the scene.

**FIGURE 5:**
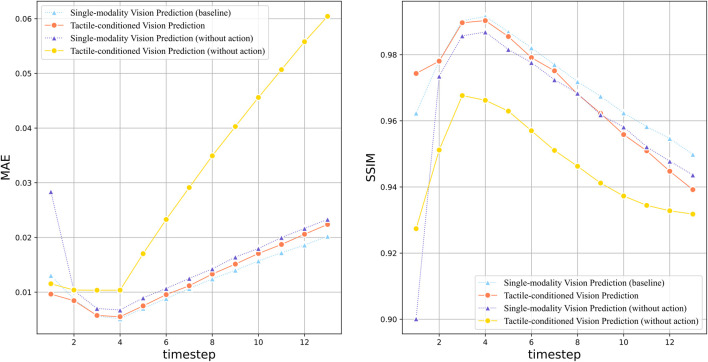
Quantitative evaluation of tactile-conditioned vision prediction over the long time horizon:the line plots illustrate the performance comparison between models with different sensation combination, including the tactile-conditioned vision prediction model (red line), single-modality baseline model (blue line), and their counterparts without action data (yellow/purple line). The graph on the left shows the MAE value of each model over 14 time steps, indicating the prediction accuracy. And the graph on the right compares the Structural Similarity Index Measure (SSIM) of the models.

Figure 5 depicted the quantitative prediction performance over long time horizon, in which the MAE and SSIM at each time step are compared between the vision prediction models with different complementary sensation. It is noteworthy that the impact of robot action data is also evaluated. Compared to sensory inputs such as vision and touch, action data reveals how specific interventions can alter the state of the environment, which is essential to gaining insight into the dynamics of cause and effect. The action-conditioned model with tactile sensation seems to perform slightly worse than its single-modality counterpart. While the two models produce results at similar level during the early stages, their performance gap increases over time. This is an intriguing outcome as tactile sensation is typically expected to enhance the model performance, especially in scenarios involving contact interactions, However, it could be reasonable due to several factors.

To start with, the interaction setting of robotic-object interaction in our experiment is relatively simple, where the motion of the system is predictable and deterministic with predefined robot action. In addition, there is no obvious slippage at the contact surface, which could impair the variability of tactile sensation that contribute to more complex manipulation tasks. Therefore, given the current setting, the visual scenes coupled with the action data specifying the movement commands could sufficiently capture the dynamics of the interaction, which dominates over the impact of tactile information.

Compared to tactile sensation, action data exert more impact on the prediction performance. It can be observed from the line plots that the single-modality vision prediction without action produces the worst outcome in terms of both MAE and SSIM, which is greatly improved by the inclusion of action data. This observation offer valuable insight into the dynamics of learning and prediction. Since action data contains direct information related to the active motion intentions of the robot, its inclusion can greatly reduce the uncertainty in prediction. In our interaction settings where the robot pushes objects in a single direction, action data can provide critical information of the intended direction, which allows the model to produce more accurate predictions.

It can be observed in both MAE and SSIM plots that the best performance of all models is achieved at time step 4, which is aligned with the results presented by Finn et al. (2016). Since the initialization of the prediction model is influenced by the utilization of context frames (which is 4 in this paper) during training, it is plausible that the contextual information enables the model to capture the short-term temporal dependencies more effectively within the range of the contextual frames, which results in the peak prediction performance.

Table 3 compares the average prediction performance of vision prediction with different sensation combination over the full prediction horizon of 14 time steps, providing an overall evaluation of the models’ capability. While the action-conditioned model outperforms other sensation conditions, the tactile-conditioned model without action performs worst in terms of both average MAE and SSIM. The comparison between single-modality vision prediction without action to its tactile-conditioned counterpart indicates that the inclusion of tactile in the absence of action guidance could introduce additional complexity instead of improvement to the prediction problem. This is in line with the aforementioned analysis that the impact of tactile sensation in simple and deterministic interactive setting could be impaired in contrast with the critical role of action data in enhancing the prediction accuracy.

**TABLE 3:** Average vision prediction performance with different sensation integration conditions.

Model	Vision prediction (without action)	Vision prediction	Tactile-conditioned vision prediction (without action)	Tactile-conditioned vision prediction
MAE ↓	0.0159	**0.0128**	0.0332	0.0135
SSIM ↑	0.9606	**0.9699**	0.9456	0.9661

Bold values indicate the best performance for each metric across the models compared.

**FIGURE 6:**
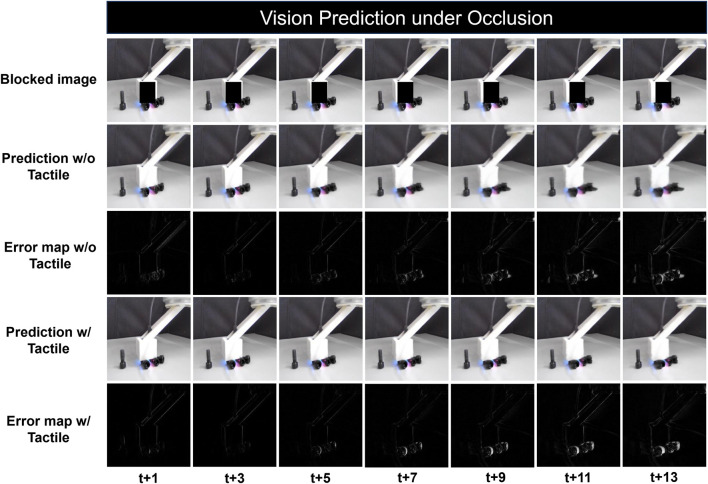
Qualitative results of vision prediction under occlusion: The first row shows the processed vision data with blocked contact area. Predictions with and without tactile sensation are shown at every three time steps from t+1 to t+13, the 3rd and 5th row shows the error map with pixel-wise difference between the prediction and the ground truth.

To further investigate the cross-modality influence between vision and touch in more specific scenarios, we conduct prediction tasks under occlusion conditions. This involves blocking the contact areas between the robot and objects in the interaction videos to simulate circumstances with limited visual information. Action data is excluded to eliminate additional influencing variables. Figure 6 presents the qualitative results over time steps, with the occluded sequences shown in the first row and pixel-wise difference maps for the tactile-enhanced and single modality models displayed in the third and fifth rows, respectively. Consistent with previous findings, an increase in blur is observed over time for both models. However, in contrast to the relatively uniform blur distribution in experiments with full vision access, the occlusion scenarios show more significant mismatches around the contact areas, highlighting the impact of restricted vision on prediction performance. Under this condition, while severe distortion occurs to the interacting objects using single modality model, the tactile enhanced model demonstrates a superior ability to restore the shapes of objects within the occluded areas, which indicates that the inclusion of tactile sensation compensates for the missing visual information and provides crucial contact details.

**FIGURE 7:**
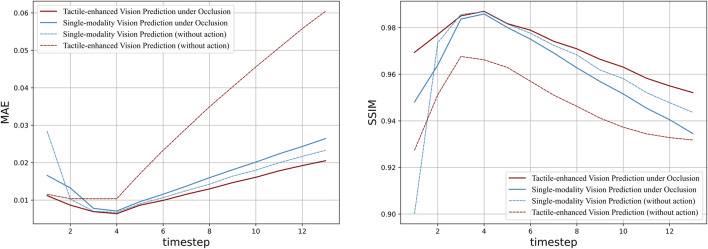
Quantitative evaluation of tactile-conditioned vision prediction under occlusion: the line plots illustrate the performance comparison between tactile-conditioned vision prediction model (red line) and single-modality baseline model (blue line), both of which excludes action data and are employed with blocked visual input. The dotted lines are their counterparts with full vision access. The graph on the left shows the MAE value of each model over 14 time steps, indicating the prediction accuracy. And the graph on the right compares the Structural Similarity Index Measure (SSIM) of the models.

The progressive quantitative results illustrated in Figure 7 highlight the complementary effect of tactile sensory input when vision is impaired. The tactile-enhanced model consistently outperforms its single-modality counterparts across the entire prediction horizon, as evidenced by both MAE and SSIM metrics. And the increasing performance gap over time aligns with the qualitative results shown in Figure 6, where the benefits of tactile sensing is more significant in the later stage of prediction, displaying robustness over long time horizon. Additionally, the model conditioned on tactile data produce better results under occlusion than with fully available vision. This surprising phenomenon indicates that while tactile sensing may bring extra noises under simple interaction settings like straight-line pushing, it plays a crucial role in robotic perception in challenging scenarios with vision loss. As found in previous research, tactile sensing can become the primary source of information when visual input is compromised, enhancing overall system perception with its high sensitivity to detecting pressure and texture variation (Zou et al., 2017). This ability of tactile sensors to provide detailed and localized feedback becomes particularly valuable in scenarios where visual data is insufficient or occluded.

On the contrary, there is performance degradation for the vision-only model when occlusion occurs, as illustrated by both MAE and SSIM curves. Vision-based models rely heavily on the availability of clear and comprehensive visual input for accurate perception and prediction. To this end, the loss of essential information about the environment and object interactions may introduce increased uncertainty, leading to limited inferring capability. While the interaction system moves as a cohesive unit in straight-line movement, there is relative motion at the contact point between the robot and objects, the deprived information of which brings difficulty to the single-modality prediction. This limitation underscores the challenges faced by single-modality visual models in scenarios where vision is impaired.

#### 3.4.2 Vision-conditioned tactile prediction

In this section, we explore the vision-conditioned tactile prediction of physical robot interaction with the inclusion of vision sensation. While the inclusion of tactile sensation into vision prediction yields negligible improvement, as discussed in previous section, the reverse scenario—enhancing tactile prediction with visual information, might be different due to the inherent modality difference between vision and touch. Hence, we investigate how visual data, characterized by its expansive and contextual information of the scene, complements the more localized tactile feedback.

**FIGURE 8:**
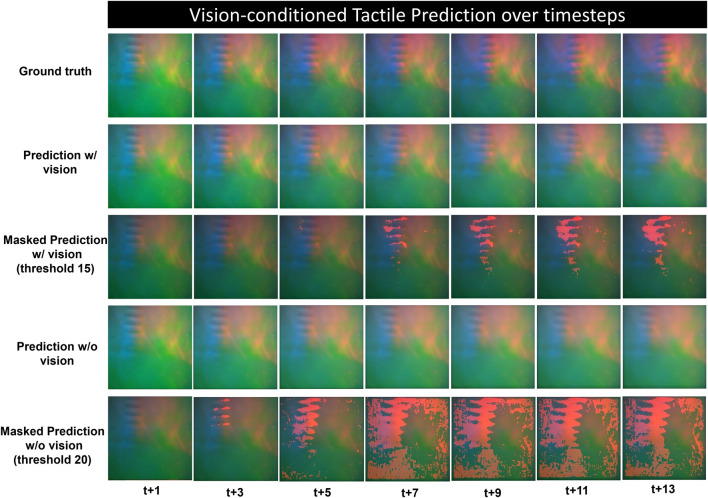
Qualitative results of vision-conditioned tactile prediction over long time horizon, the ground truth and predicted frames are shown at every three time steps from t+1 to t+13, the 3rd and 5th row shows the predictions masked with pixel-wise difference between the prediction and the ground truth above a specific threshold.

Figure 8 shows the qualitative prediction results over time, comparing the action-conditioned tactile prediction model with and without the inclusion of vision sensation. The vision-based tactile sensor produced tactile images that visualize the deformation of contact surface which locates the contact position of the interaction objects. Rows 3 and 5 of the figure display the predicted frames overlaid with a mask representing the discrepancy from the ground truth. This mask highlights areas where the prediction deviates from the actual frame by a pixel value exceeding a specified threshold.

The result indicates a decline in prediction accuracy for both models over time, characterized by blurring and a lag in object position relative to the ground truth. However, incorporating visual data significantly enhances the prediction performance, especially in terms of the robustness across extended time sequences. An analysis of the progressively depicted predictions in rows 2 and 4 of Figure 8 reveals that the model augmented with visual information generates more plausible predictions that more closely align with the ground truth both in terms of clarity and color saturation. In addition, to facilitate observation, the predictions of both models have been masked according to varying pixel-wise error thresholds. In the initial phase of the time horizon, while the vision-enhanced model yields highly accurate prediction with negligible masked error over the threshold of 15 pixel value (15 out of 255), significant errors are evident in the vision-only predictions with a higher threshold of 20. As time progresses, the discrepancy between the vision-enhanced prediction and the ground truth mainly manifest at the leading edge of moving features. In contrast, the masked error in non-tactile vision prediction become more widely-distributed, affecting the entire scope of the tactile image.

**FIGURE 9:**
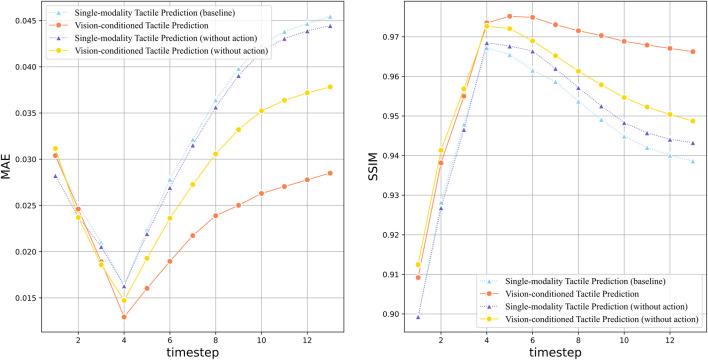
Quantitative evaluation of vision-conditioned tactile prediction over long time horizon: the line plots illustrate the performance comparison between models with different sensation combination, including the vision-conditioned tactile prediction model (red line), single-modality baseline model (blue line), and their counterparts without action data (yellow/purple line). The graph on the left shows the MAE value of each model over 14 time steps, indicating the prediction accuracy. And the graph on the right compares the Structural Similarity Index Measure (SSIM) of the models.

Figure 9 depicted the quantitative prediction performance over long time horizon, in which the MAE and SSIM at each time step are compared between the tactile prediction models with different complimentary sensation. The vision-enhanced tactile prediction model yields a superior prediction performance than other models in terms of both MAE and SSIM, which indicates that the integration of visual information plays a pivotal role in enhancing the quality of tactile predictions. In addition, the vision-enhanced model also demonstrates outstanding long-term prediction stability, exhibiting milder degradation in performance across the extended temporal horizon.In contrast, the MAE for the other models escalates sharply with time, underlining the enhanced reliability of the vision-enhanced approach. Similarly, the vision-enhanced tactile prediction model rapidly achieves SSIM scores above the 0.95 threshold, presenting efficient prediction in the short term. For long-term prediction, the slower declining rate also indicates that visual information contributes significantly to maintaining structural fidelity over time.

The single-modality tactile prediction with action data produces worse predictions compared to its counterpart excluding the action data. However, when combined with visual sensation, the action-conditioned model performs better than the non-action one. This may be caused by the role of visual data in providing a broader contextual understanding of the interactive scene that tactile sensation lacks. In physical interaction tasks, the visual modality captures the spatial dynamics and interactions that are crucial for interpreting the implications of actions. When tactile prediction models are deprived of this context, action data alone would be insufficient to accurately anticipate future states. Instead, it may lead to an overemphasis on the direct physical consequences of actions, without accounting for the larger environmental context, resulting in poorer predictions. Conversely, when visual information is present, it can effectively contextualize the action data, enabling the model to make more informed predictions about how actions will affect the tactile sensory inputs. This suggests a synergistic interplay between vision and action data in tactile prediction, where vision provides the necessary framework for action data to be meaningfully incorporated.

Table 4 compares the average prediction performance of tactile prediction with different sensation combination over the full prediction horizon of 14 time steps, in which the vision-enhanced tactile prediction model outperforms the tactile-only prediction model either with or without action data in both evaluation metrics. And the action-conditioned model enhanced by vision yields the lowest MAE and Highest SSIM, which suggests its overall prediction’s high structural and textural consistency with the ground truth.

**TABLE 4:** Average tactile prediction performance with different sensation integration conditions.

Model	Tactile prediction (without action)	Tactile prediction	Vision-conditioned tactile prediction (without action)	Vision-conditioned tactile prediction
MAE ↓	0.0330	0.0339	0.0291	**0.0237**
SSIM ↑	0.9479	0.9453	0.9544	**0.9626**

Bold values indicate the best performance for each metric across the models compared.

In conclusion, the inclusion of vision sensation into tactile prediction models is crucial to improves the system’s cause-effect understanding and perception during physical robot interaction. And a cross-modality influence has been found between vision and action in tactile prediction, indicating the multiple sensations are deeply intertwined with the environment dynamics during interaction.

## 4 Discussion

It has been commonly acknowledged that humans rely on the predictive learning of multi-modal sensorimotor signals to perform complex interaction tasks in the dynamic physical world, this is bionically based on the nature of human cognition where sensations such as vision and touch are highly intertwined. In this work, we aim to explore the synergy and cross-modality influence between vision and tactile sensory inputs in robotics. Our goal is to enhance the predictive learning of physical robot interaction through the integration of multiple sensing modalities, which is essential for developing the next-generation of intelligent robotic systems with more advanced perception and manipulation capabilities in the complex real-world setting. To this end, we introduce a multi-modal fusion mechanism into the well-known video prediction framework Convolutional Dynamic Neural Advection (CDNA) (Finn et al., 2016), based on which we conduct a series of interaction prediction experiments involving robot-object interaction tasks using different combination of sensory input. The quantitative and qualitative evaluation of the prediction performance provide valuable insights into the connection between vision and touch in the robot’s understanding of cause-and-effect relationship during physical interaction. In addition, the scope of this work is not restricted within vision and touch like other related studies, but is extended to include action data that reveals the system dynamics into investigation. This inclusion of action distinguishes our work from static analysis of vision-tactile fusion, uncovering the complexity of dynamics underlying the motion.

The quantitative and qualitative results of tactile prediction with different sensory combination reveal several invaluable insights into the synergy between vision, tactile and action inputs. The inclusion of visual sensation into action-conditioned tactile prediction yields superior performance to other models, which underscores the significance of visual sensing in providing broader scene comprehension that complement the more localized contact information of tactile data. In addition, the synergy between vision and action has been uncovered by a seemingly counter-intuitive observation, where the action data fails to enhance the performance of tactile prediction in the absence of visual inputs. This entanglement between action and vision is comprehensible as the vision data provides the contextual information of the overall interaction scene that complement the interpretation and utilization of action data for the robotic perception system. Therefore, our enhanced multi-modal prediction model of physical robot interaction successfully develops the inner connection between vision, tactile and action, which aligns with human cognition, opens up new possibilities towards more comprehensive robotic perception systems that enable more intelligent and adaptive interaction with the environment.

Alternatively, the results of vision prediction provide a more complicated scenario, where the inclusion of tactile sensation makes negligible impact on the predictability of the model, producing outcomes even slightly less accurate than the single-modality vision prediction model. There is reasonable possibility that the simple interaction setting of straight-line robot-object interaction account for the impaired significance of tactile data, especially in presence of action data that provide more direct motion information. While the tactile sensation excels in offering necessary contact information during complex manipulation tasks such as in-hand manipulation, in current tasks, the lack of complex relative movement between the robot and object restricts its efficient involvement in prediction. The experiments with occluded vision verify this explanation to some extent, in which the introduction of impaired vision around critical contact area brings different results. While the vision model produces distorted inference over time, the tactile-enhanced model demonstrated superior performance under occlusion conditions. The integration of tactile sensory input compensates for the loss of visual data, providing detailed and localized feedback about the object’s properties and interactions. Tactile sensors are highly sensitive to variations in pressure and texture, enabling the model to maintain accurate predictions even when visual information is partially or entirely occluded. This sensitivity is particularly advantageous in scenarios where precise physical interactions are critical, such as tool-use in medical applications. Additionally, action data also plays a crucial role in enhancing the prediction accuracy in scene estimation, which reflects the synergetic effect of integrating environment dynamics with overall visual context.

It is noteworthy that the asymmetric results between vision and tactile prediction implies the inherent modality difference between vision and touch, despite the influence of experiment setting. Vision provides broader contextual information from a global perspective, which facilitates the anticipation of interactions across the entire observed scene. Tactile sensing, in contrast, yields high-resolution local contact information critical to immediate interaction details, such as texture, pressure, and temperature. Therefore, in the scenario of interaction prediction, while vision can easily complement tactile with its rich spatial information, tactile sensing may not always enhance vision predictions to the same extent. This limitation can arise because tactile data, with its focus on immediate, localized contact information, may not provide additional predictive value for the broader, visually observable dynamics of an interaction. However, tactile can complement the limitations of vision in certain case, such as self-occlusion in manipulation and control tasks with limited light resource. It is estimable that with more efficient multi-modal fusion mechanism, the integration of tactile sensation can lead to more adaptive and dexterous manipulation system. Therefore, in future work, more generalised and effective multi-modal perception system should be developed to accommodate efficient cross-modal integration.

## 5 Conclusion

In this work, we presented an enhanced predictive model of physical robot interaction that achieves integration of vision and tactile sensation, aiming to investigate the connection between multiple sensory input. To evaluate the model, we developed a robotic system for object interactive tasks, which is equipped with comprehensive sensory inputs, including recorded visual scene and vision-based tactile sequences. A robot-object interaction dataset containing 250 trials with different object was created to store synchronized data of vision, tactile feedback, and robot actions. Through both qualitative and quantitative analysis, the proposed vision-enhanced tactile prediction model produced promising result that underscores the cross-modality influence between different sensation. And the superior performance of tactile-conditioned vision prediction model under partial occlusion reveals the complementary effect between vision and touch. Additionally, the role of action in complementing prediction performance was also evaluated.

Our thorough analysis provides insightful information regarding the nature of interdependence and modality differences between vision and touch. Consequently, this work opens the possibilities towards more intelligent robotic perception system that narrows the gap between artificial agents and human recognition.

In future work, the proposed model could be integrated with state-of-the-art control mechanisms such as reinforcement learning, which enables more adaptive manipulation in complex and dynamic physical settings through efficient multi-modal representation. Furthermore, the promising results in tactile prediction open avenues for future research in physical interaction, such as tool-use in robotic surgery, automation in healthcare, etc. In conclusion, our work demonstrates the potential of multi-modal sensory fusion in enhancing the robotic perception during physical interaction, which presents promising prospect for innovation and application in diverse fields.

## Data Availability

The raw data supporting the conclusions of this article will be made available by the authors, without undue reservation.
